# Effect of a multicomponent intervention on institutionalization-free survival in older adults with sarcopenia: a *post-hoc* analysis

**DOI:** 10.3389/fmed.2025.1573384

**Published:** 2025-09-03

**Authors:** Sunghwan Ji, Eunju Lee, Ji Yeon Baek, Geon Young Jang, Hee-Won Jung, Il-Young Jang

**Affiliations:** ^1^Department of Digital Health, Samsung Advanced Institute for Health Sciences and Technology, Sungkyunkwan University, Gangnam-gu, Republic of Korea; ^2^Asan Medical Center, College of Medicine, University of Ulsan, Songpa-gu, Republic of Korea

**Keywords:** sarcopenia, multicomponent intervention, physical frailty, frailty, public health

## Abstract

**Objectives:**

To assess the effect of a 24-week comprehensive multicomponent intervention on institutionalization-free survival, physical performance, and frailty among older adults with sarcopenia.

**Design:**

A post-hoc analysis of a prospective, non-randomized intervention study with 1:1 propensity score matching.

**Setting:**

Community-dwelling, socioeconomically vulnerable older adults.

**Participants:**

A total of 283 older adults with sarcopenia were included, with 145 in the intervention group and 138 in the control group. After propensity score matching, 102 pairs were analyzed. The mean age was 77.57 years (intervention) and 77.64 years (control), with 82.4 and 81.4% females in each group, respectively.

**Intervention:**

The multicomponent intervention consisted of exercise, nutritional support, depression management, deprescribing, and home hazard reduction, implemented over 24 weeks.

**Measurements:**

The primary outcome was 30-month institutionalization-free survival. Secondary outcomes included changes in physical performance (Short Physical Performance Battery [SPPB] scores, gait speed) and frailty index over 6, 18, and 30 months.

**Results:**

Following propensity score matching, mortality and institutionalization occurred in 13 (12.7%) and 35 (34.3%) participants in the intervention and control groups, respectively. A significant difference in 30-months institutionalization-free survival was observed between the intervention and control groups (63.4% vs. 87.2%). The intervention group had significantly higher SPPB scores and improved gait speed at 6 months, 18 months, and 30 months. The intervention group showed a significantly lower frailty index only at 6 months but similar scores at 18 and 30 months.

**Conclusion:**

The multicomponent intervention significantly improved long-term institutionalization-free survival and physical function in older adults with sarcopenia, highlighting its potential to enhance independence and reduce frailty in vulnerable populations.

## 1 Introduction

Sarcopenia, a progressive loss of skeletal muscle mass and function, is a highly prevalent geriatric syndrome linked to adverse outcomes such as falls, disability, hospitalization, institutionalization, and mortality (1–3). Its global prevalence is estimated to range from 10% to over 20% among community-dwelling older adults, depending on diagnostic criteria and population characteristics (2). Despite its clinical significance, intervention strategies are limited, with most guidelines only recommending exercise and nutritional support (4–7). Therefore, there is an urgent need to explore more effective interventions that can address needs of older adults with sarcopenia.

Frailty, another common aging-related syndrome, is characterized by increased vulnerability to stressors due to multisystem decline (8). Sarcopenia and frailty frequently coexist and share overlapping biological mechanisms—such as chronic inflammation, hormonal dysregulation, malnutrition, and physical inactivity—that contribute to impaired physical function and resilience (9, 10). Numerous longitudinal studies have demonstrated that both conditions are similarly associated with adverse outcomes, including, institutionalization, and mortality (11–13). A recent systematic review and meta-analysis further showed that several biomarkers, such as serum albumin and hemoglobin, are commonly implicated in both frailty and sarcopenia, reinforcing their biological convergence (14). In fact, some researchers suggest that sarcopenia and frailty may be difficult to disentangle, as they often coexist and manifest through similar clinical pathways (15, 16). Therefore, management strategies developed for frailty—particularly multicomponent geriatric interventions—may also be effective for sarcopenia.

Frailty management involves patient-centered multicomponent geriatric intervention, addressing unmet needs comprehensively (17, 18). These interventions extend beyond exercise and nutritional support to include deprescribing, mental health management, and environmental modifications—emphasizing the underlying causes of inactivity and anorexia in older adults (17). The World Health Organization’s Integrated Care for Older People (ICOPE) framework exemplifies these principles (19), and several geriatric care guidelines have incorporated this holistic approach (20, 21). Although sarcopenia and physical frailty substantially overlap, the specific effectiveness of multicomponent geriatric interventions on sarcopenia outcomes remains insufficiently established. This challenge arises from the very nature of geriatric care, which is inherently individualized and multifactorial—making it difficult to evaluate with traditional disease-specific trial designs (22). In this context, conducting a post-hoc analysis of sarcopenic subgroups within previously conducted frailty intervention trials is a meaningful and justified approach to explore potential benefits in this high-risk population.

The Aging Study of Pyeongchang Rural Area-Intervention Study (ASPRA-IS) study, a non-randomized clinical trial, was designed to assess the effectiveness of a 24-week multicomponent intervention in socially vulnerable older adults living in the community, with the results detailed in a prior study. In summary, prior studies demonstrated that the program reduced the risk of disability (23), institutionalization-free survival over 30 months (24), by improvements in physical performance (25). The intervention included various components, including exercise, nutrition, depression management, deprescribing, and home hazard reduction (25). In this study, we conducted a *post hoc* analysis specifically examining participants with sarcopenia within the ASPRA-IS to determine whether the multicomponent intervention is effective in older adults with sarcopenia. Furthermore, we examined its effectiveness across multiple operational definitions of sarcopenia—including those defined by the Asian Working Group for Sarcopenia (AWGS) (4), the Korean Working Group on Sarcopenia (KWGS) (26), as well as subtypes such as severe sarcopenia and functional sarcopenia.

## 2 Methods

### 2.1 Study design

This study was a *post hoc* analysis of the ASPRA-IS, a prospective, single-arm intervention study that conducted a 24-week multicomponent intervention in Pyeongchang County, Gangwon Province, South Korea. Details of the ASPRA-IS study are described in previous studies (24, 25). In summary, ASPRA-IS enrolled participants who lived alone or received medical aid from the ASPRA cohort, an ongoing prospective cohort study of community-dwelling older adults (27). Exclusion criteria included inability to walk 100 m, recent admission to long-term care facilities, diagnoses of end-stage heart failure, end-stage renal disease, metastatic cancer, cognitive impairment (Mini-Mental State Examination score ≤ 18 points), and plans to relocate outside the study area within the next 6 months (27).

From the ASPRA cohort, 383 eligible individuals were identified, with 187 opting for the multicomponent intervention and 196 choosing not to participate. Since those who declined intervention underwent the same comprehensive geriatric assessment as part of the observational cohort (ASPRA cohort), information was collected for both the intervention and control groups. The study protocol received approval from the Institutional Review Boards of Asan Medical Center and was registered in 2015 (NCT02554994). Informed consent was obtained from all participants before the entry. This study was performed in accordance with the ethical standards laid down in the 1964 Declaration of Helsinki and its later amendments.

### 2.2 Study population and assessment of sarcopenia

Among 383 participants of the ASPRA-IS study, participants with sarcopenia were the focus of this investigation. Sarcopenia was defined in accordance with the AWGS (4) and KWGS guidelines (26). Bioelectrical impedance analysis (BIA) was employed to measure muscle mass at frequencies of 5, 50, and 500 kHz. Appendicular skeletal mass (ASM) was calculated by summation of the lean mass of both arms and legs, adjusted by height squared (ASM/h^2^). Low muscle mass was identified as ASM/h^2^ below 7.0 kg/m^2^ in men and below 5.7 kg/m^2^ in women, measured after an overnight fast. Grip strength was assessed using a handgrip dynamometer (T. K. K 5401 Grip-D; Takei, Tokyo, Japan), with low grip strength defined as <28 kg for men and <18 kg for women. Usual gait speed was determined by instructing participants to walk 7 m at their regular pace on a flat indoor surface. Trained nurses measured the 4 m transit time with a digital stopwatch, excluding the acceleration and deceleration interval of 1.5 m. Slow gait speed was defined as <1 m/s.

Severe sarcopenia was identified in individuals exhibiting low muscle mass, low muscle strength, and slow gait speed. Sarcopenia (not severe) was defined as low muscle mass with either low muscle strength or slow gait speed, not meeting the criteria for severe sarcopenia. Functional sarcopenia was defined as having low muscle strength and slow gait speed without low muscle mass. This definition was introduced in the KWGS guidelines (26) and has been validated in previous studies regarding its comparable prognosis with earlier sarcopenia definitions (13) and its response to exercise and nutritional interventions (28). Among the 285 participants with sarcopenia, 2 with missing variables [Center for Epidemiologic Studies Depression (CES-D) scale] were excluded, resulting in 283 participants for propensity score matching. Of these, 138, 42, and 103 participants were classified as having severe sarcopenia, sarcopenia (not severe), and functional sarcopenia, respectively. Additionally, 145 and 138 participants were assigned to the intervention and control groups, respectively. The study flow chart is outlined in Supplementary Figure S1.

### 2.3 A multicomponent intervention

The 24-week multicomponent intervention program comprised group exercise, nutritional supplementation, depression management, medication review, and home hazard reduction. A detailed description of the intervention is available in a prior study (24, 25). In brief, all participants received group exercise sessions lasting 60 min twice a week, along with commercial nutritional supplements (125 mL liquid formula containing 200 kcal, 24.5 g carbohydrate, 13 g protein, 5.63 g essential amino acid, and 7 g fat) twice daily (29–31). The depression management program (32), deprescribing for potentially inappropriate medications for older adults (33), and home hazard evaluation and reduction were selectively administered to eligible participants based on predefined criteria (Table 1).

**Table 1:** Overview of the multicomponent intervention program.

Focus	Description of intervention
Exercise	• Intervention: 60-min group exercise session led by licensed trainers focusing on the following types. The intensity started with low-intensity exercises and increased intensity every month 1. Resistance (20 min): squat, plank, side plank, straight leg raises 2. Balance (20 min): one-leg standing, shifting from side to side, heel-to-toe walk 3. Aerobic/endurance (20 min): step up and down, quick pace, dancing 4. The exercise trainer was given instructions not to exceed 60–70% of the maximal exercise capacity based on the perceived exertion scale • Target: all participants • Frequency: twice a week
Nutrition	• Intervention: administration of 125 mL commercial liquid formula containing 200 kcal of energy, 24.5 g carbohydrate, 13 g protein, 5.63 g essential amino acid, and 7 g fat • Target: all participants • Frequency: twice a day
Depression	• Intervention: evaluation by a geriatrician or a psychiatrist and administration of supportive psychotherapy or antidepressant medication as clinically indicated • Target: participants with a CES-D score > 20 points at baseline • Frequency: monthly
Polypharmacy	• Intervention: medication review by a geriatrician, and dose reduction or discontinuation of potentially inappropriate medications according to the 2012 Beer’s criteria • Target: participants taking five prescription medications at baseline • Frequency: monthly
Home hazards	• Intervention: evaluation of home environment by a visiting nurse and a social worker using the Home Fall Prevention Checklist by the Centers for Disease Control and Prevention and modification of the environment to eliminate any identified hazard • Target: all participants with any identified home hazard at baseline • Frequency: trimonthly

CES-D, center for epidemiologic studies depression.

Source: Oh G, Lee H, Park CM, Jung H-W, Lee E, Jang I-Y, et al. Long-term effect of a 24-week multicomponent intervention on physical performance and frailty in community-dwelling older adults. Age and Ageing. 2021; 50: 2157–66.

The 24-week multicomponent intervention was implemented 6 months after the baseline assessment (−6 months). Throughout the 6-month pre-intervention period, participants received routine care from local public health centers. After completing the 24-week multicomponent program, the intervention group transitioned to receiving routine care, serving as the comparison group. Adherence rates ranged from 83.7 to 91.3% across each subtype of the intervention program (23). Meanwhile, the control group continued to receive routine care throughout the entire study period.

### 2.4 Comprehensive geriatric assessment

Comprehensive geriatric assessment was performed every year: at baseline (6 months before the start of the intervention program), 6 months (at the end of the intervention), 18 and 30 months. Trained nurses who were unaware of the intervention status performed comprehensive geriatric assessment. For the intervention group, additional comprehensive geriatric assessment was performed at the start of the intervention program (0 months).

Data were collected on demographic characteristics, years of completed education, and identification of individuals with low socioeconomic status (those receiving medical aid due to a monthly income of <500 USD). Chronic conditions were collected, including 11 physician-diagnosed clinical conditions (angina, arthritis, asthma, cancer, chronic lung disease, congestive heart failure, diabetes, heart attack, hypertension, kidney disease, and stroke). Depressive symptoms were evaluated using the Korean version of the CES-D Scale (34). Cognitive status was assessed using the Mini-Mental State Examination for Dementia Screening (35). The risk of malnutrition was determined using the Mini-Nutritional Assessment Short Form score, with a score of ≤ 11 indicating malnutrition risk (36).

### 2.5 Outcome assessment

Institutionalization-free survival served as the outcome measure, assessed at 3-month intervals by nursing staff. The occurrence month and reasons for loss to follow-up were obtained directly from study participants or their family members. Changes in the Short Physical Performance Battery (SPPB) score (ranging from 0 to 12 points and encompassing usual gait speed, standing balance, and completion of five chair stands), usual gait speed, and a 47-item Frailty Index were also evaluated. The 47-item Frailty Index was calculated based on the deficit-accumulation theory using 47 specified items (Supplementary Table 1) (37).

### 2.6 Statistical analysis

We conducted 1:1 propensity score matching using a nearest-neighbor method with a caliper width of 0.2 standard deviation of the logit propensity score. The propensity score model was developed using logistic regression, with intervention status specified as the dependent variable. Baseline characteristics, including age, sex, enrolled year, living alone, CES-D score, number of chronic diseases, number of falls in the last year, emergency room or admission in the previous year, frailty phenotype, frailty index, gait speed, and sarcopenia phenotype, were used as independent variables. The balance in baseline characteristics between the two groups was assessed using standardized mean difference (SMD).

The SPPB score of 70 participants in this investigation was not measured due to a protocol update after the baseline assessment of participants enrolled in 2014. The SPPB score was imputed in the pre-matching cohort using mice R package with baseline gait speed, SPPB score at baseline and 0 months (intervention group) and 6 months (control group) among participants enrolled excluding 2014.

We summarized the mean and standard deviation or proportions of baseline characteristics for both groups before and after propensity score matching. A linear mixed model with random intercept was used to determine the effect of the intervention on the SPPB score, gait speed, and frailty index at 6, 18, and 30 months. This model included independent variables for intervention status, times as categorical variables, and their interaction terms. The mean differences (MDs) in SPPB score, gait speed, and frailty index between the two groups at 6, 18, and 30 months and their 95% confidence intervals (CI) were calculated from a linear mixed model.

Institutionalization-free survival was determined using Kaplan–Meier estimates. To examine the statistical differences in survival and hazard between the intervention and comparison groups, we employed the log-rank test and Cox proportional hazard model. Additionally, a subgroup analysis was performed by categorizing participants into two groups based on the presence or absence of low muscle mass, classified as sarcopenia (AWGS) and functional sarcopenia.

To underscore the robustness of the association between intervention status and institutionalization-free survival, a sensitivity analysis was conducted after categorizing participants into four groups: (A) individuals with low muscle mass, slow gait speed, and low grip strength (severe sarcopenia); (B) individuals with low muscle mass and slow gait speed but preserved grip strength; (C) individuals with low muscle mass and low grip strength but preserved gait speed; and (D) individuals with slow gait speed and low grip strength but preserved muscle mass (functional sarcopenia). We compared the institutionalization-free survival of the intervention and control groups across various combinations of A, B, C, and D.

A two-sided *p*-value <0.05 significance threshold was applied for all analyses to determine statistical significance. Statistical analyses were performed with R Software (version 4.1.1; R Foundation for Statistical Computing, Vienna, Austria).

## 3 Results

### 3.1 Baseline characteristics

In comparing the intervention (*N* = 145) and control (*N* = 138) groups, the intervention group had a higher mean age (77.8 years) than the control group (76.9 years). The percentage of females was higher in the intervention group (77.2%) than in the control group (75.4%). Grip strength was lower in the intervention group (15.2 kg) than in the control group (16.7 kg). Additionally, the intervention group had a lower risk of depression (mean CES-D score 10.3 versus 11.5). The prevalence of falls in the last year was higher in the intervention group (22.1%) compared to the control group (15.2%). Furthermore, the intervention group showed a higher number of chronic conditions (1.7 versus 1.5), slower gait speed (0.62 versus 0.66 m/s), worse SPPB score (6.97 versus 7.56), and greater frailty, as indicated by higher scores on both the frailty phenotype scale (2.6 versus 2.2) and frailty index (0.28 versus 0.26) than the control group.

Propensity score matching yielded 102 pairs, achieving effective balance in baseline characteristics between the two groups, as evidenced by absolute values consistently <0.1. Key variables, such as age (77.57 versus 77.64), percentage of females (82.4% versus 81.4%), and frailty index (0.27 versus 0.28), demonstrated appropriate balances (Table 2). Furthermore, variables related to the multicomponent intervention, such as the number of medications (2.91 versus 3.01), CES-D score (10.76 versus 10.90), and the percentage of individuals with fall event for the last year (17.6% versus 15.7%) also demonstrated appropriate balances.

**Table 2:** Comparison of baseline characteristics before and after propensity score matching.

Baseline characteristics	Before matching			After matching		
	Intervention (*N* = 145)	Control (*N* = 138)	SMD	Intervention (*N* = 102)	Control (*N* = 102)	SMD
Age, mean (SD)	77.78 (4.76)	76.91 (6.60)	0.151	77.57 (4.76)	77.64 (6.77)	0.012
Female, *n* (%)	112 (77.2)	104 (75.4)	0.044	84 (82.4)	83 (81.4)	0.025
Enrolled year, *n* (%)			0.197			0.027
2014	39 (28.3)	29 (20.0)		19 (18.6)	20 (19.6)	
2015	50 (36.2)	61 (42.1)		42 (41.2)	42 (41.2)	
2016	49 (35.5)	55 (37.9)		41 (40.2)	40 (39.2)	
Sarcopenia phenotype, *n* (%)			0.199			0.064
Severe sarcopenia	76 (52.4)	62 (44.9)		56 (54.9)	54 (52.9)	
Sarcopenia (not severe)	17 (11.7)	25 (18.1)		13 (12.7)	12 (11.8)	
Functional sarcopenia	52 (35.9)	51 (37.0)		33 (32.4)	36 (35.3)	
Medical aid, *n* (%)	31 (21.4)	27 (19.6)	0.045	19 (18.6)	21 (20.6)	0.049
Living alone, *n* (%)	120 (87.0)	117 (80.7)	0.171	87 (85.3)	87 (85.3)	<0.001
ASM/height^2^, kg/m^2^, mean (SD)	5.70 (1.15)	5.73 (1.00)	0.029	5.67 (1.06)	5.58 (1.00)	0.084
Grip strength, kg, mean (SD)	15.22 (5.79)	16.69 (6.90)	0.232	15.36 (5.63)	15.33 (6.13)	0.005
No. chronic conditions, mean (SD)	1.66 (1.08)	1.46 (1.05)	0.186	1.48 (1.11)	1.55 (1.07)	0.063
No. medications, mean(SD)	3.48 (3.52)	2.95 (3.11)	0.158	2.91 (2.66)	3.01 (3.16)	0.034
CES-D score, mean (SD)	10.26 (9.83)	11.48 (10.50)	0.120	10.76 (10.26)	10.90 (10.07)	0.014
MMSE-DS score, mean (SD)	23.67 (4.02)	23.76 (4.70)	0.022	23.30 (4.16)	23.27 (5.12)	0.008
Emergency room visit or admission in the last year, *n* (%)	27 (18.6)	22 (15.9)	0.071	13 (12.7)	15 (14.7)	0.057
Fall in the last year, *n* (%)	32 (22.1)	21 (15.2)	0.177	18 (17.6)	16 (15.7)	0.053
SPPB total score, mean (SD)	6.97 (2.56)	7.56 (2.76)	0.220	7.11 (2.42)	7.14 (2.80)	0.011
Gait speed, m/s, mean (SD)	0.62 (0.20)	0.66 (0.21)	0.205	0.63 (0.19)	0.62 (0.20)	0.020
Frailty phenotype, mean (SD)	2.57 (1.08)	2.24 (1.09)	0.301	2.42 (1.04)	2.45 (0.99)	0.029
Frailty index, mean (SD)	0.28 (0.09)	0.26 (0.11)	0.161	0.27 (0.09)	0.27 (0.11)	0.054

ASM, appendicular skeletal mass; CES-D, center for epidemiologic studies depression; SMD, standardized mean difference; SPPB, short physical performance battery; MMSE-DS, mini-mental state examination for dementia screening.

### 3.2 Outcomes

**Figure 1:**
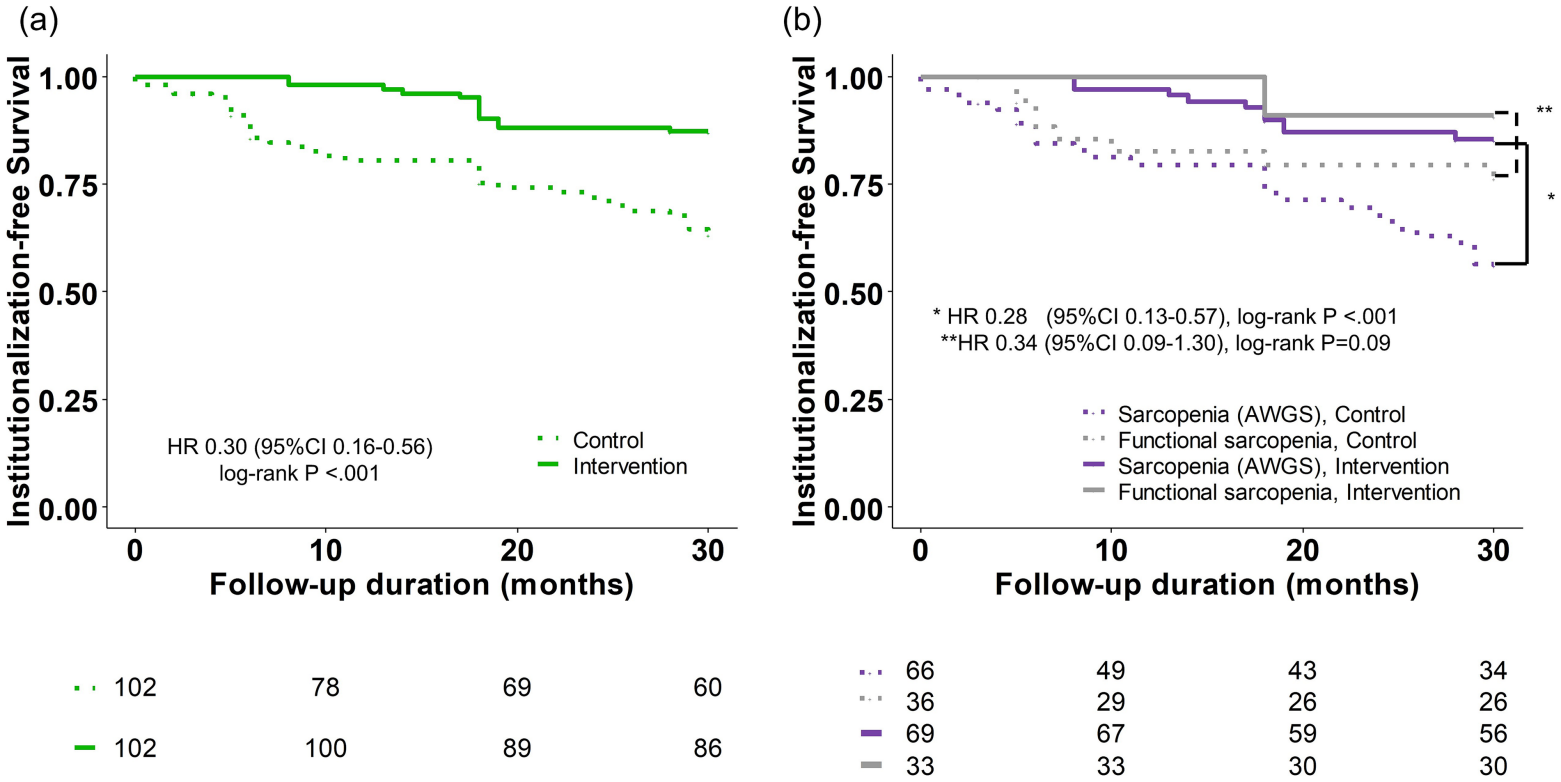
Kaplan–Meier estimate of institutionalization-free survival. **(a)** Total population. **(b)** Subgroup analysis according to Sarcopenia (AWGS) and functional sarcopenia. AWGS, Asian Working Group for Sarcopenia; CI, confidence interval; HR, hazard ratio.

In the matched cohort, mortality and institutionalization incidence were 6 (5.9%) and 7 (6.9%) in the intervention group and 12 (11.8%) and 23 (22.5%) in the control group, respectively (Detailed reasons for follow-up loss are described in Supplementary Figure S1). The 30-month institutionalization-free survival was 63.4% (95% CI, 54.4–73.9%) in the intervention group and 87.2% (81.0–94.0%) in the control group. A significant difference in institutionalization-free survival was observed between the intervention and control groups (log-rank *p* < 0.001), with a hazard ratio of 0.30 (95% CI, 0.16–0.56) (Figure 1a).

Additionally, a subgroup analysis was conducted by dividing the participants into two subgroups: those with and without low muscle mass (classified as sarcopenia (AWGS) and functional sarcopenia) (Figure 1b). In both subgroups, institutionalization-free survival maintained a noticeable difference between the intervention and control groups, although statistical significance was not reached in the functional sarcopenia group (log-rank *p* < 0.001 and *p* = 0.09, respectively). The hazard ratios for these comparisons were 0.28 and 0.34 (95% CI, 0.13–0.57, and 0.09–1.30), respectively.

**Figure 2:**
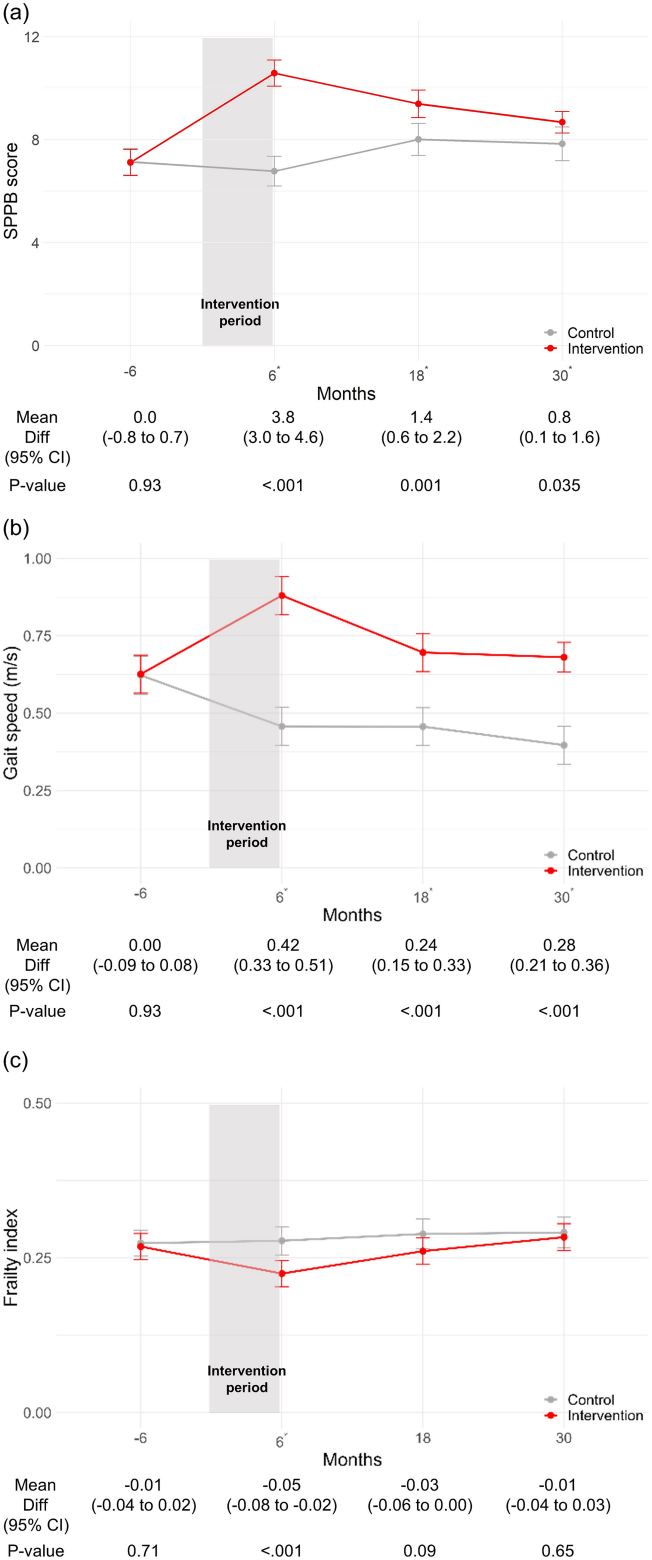
Change in SPPB score, gait speed, and frailty index stratified by intervention status. **(a)** Change in SPPB score. **(b)** Change in gait speed (m/s). **(c)** Change in frailty index. *p-value <0.05. CI, confidence interval; SPPB, short physical performance battery.

Furthermore, when examining specific outcomes, the intervention group demonstrated significantly higher SPPB scores than the control group at 6 months (MD 3.8; 95% CI, 3.0–4.6; *p* < 0.001), 18 months (1.4; 95% CI, 0.6–2.2; *p* = 0.001), and 30 months (0.8; 95% CI, 0.1–1.6; *p* = 0.035) (Figure 2a). Moreover, the intervention group showed faster gait speed than the control group at 6 months (MD 0.42; 95% CI, 0.33–0.51; *p* < 0.001), 18 months (0.24; 95% CI, 0.15–0.33; p < 0.001), and 30 months (0.28; 95% CI, 0.21–0.36; p < 0.001) (Figure 2b). Lastly, the intervention group had a significantly lower frailty index only at 6 months (MD -0.05; 95% CI, −0.08 to −0.02; p < 0.001) and lower but without statistical significance at 18 months (MD -0.03; 95% CI, −0.06 to 0.00; *p* = 0.09) and 30 months (MD -0.01; 95% CI, −0.04 to 0.03; *p* = 0.65) (Figure 2c).

**Figure 3:**
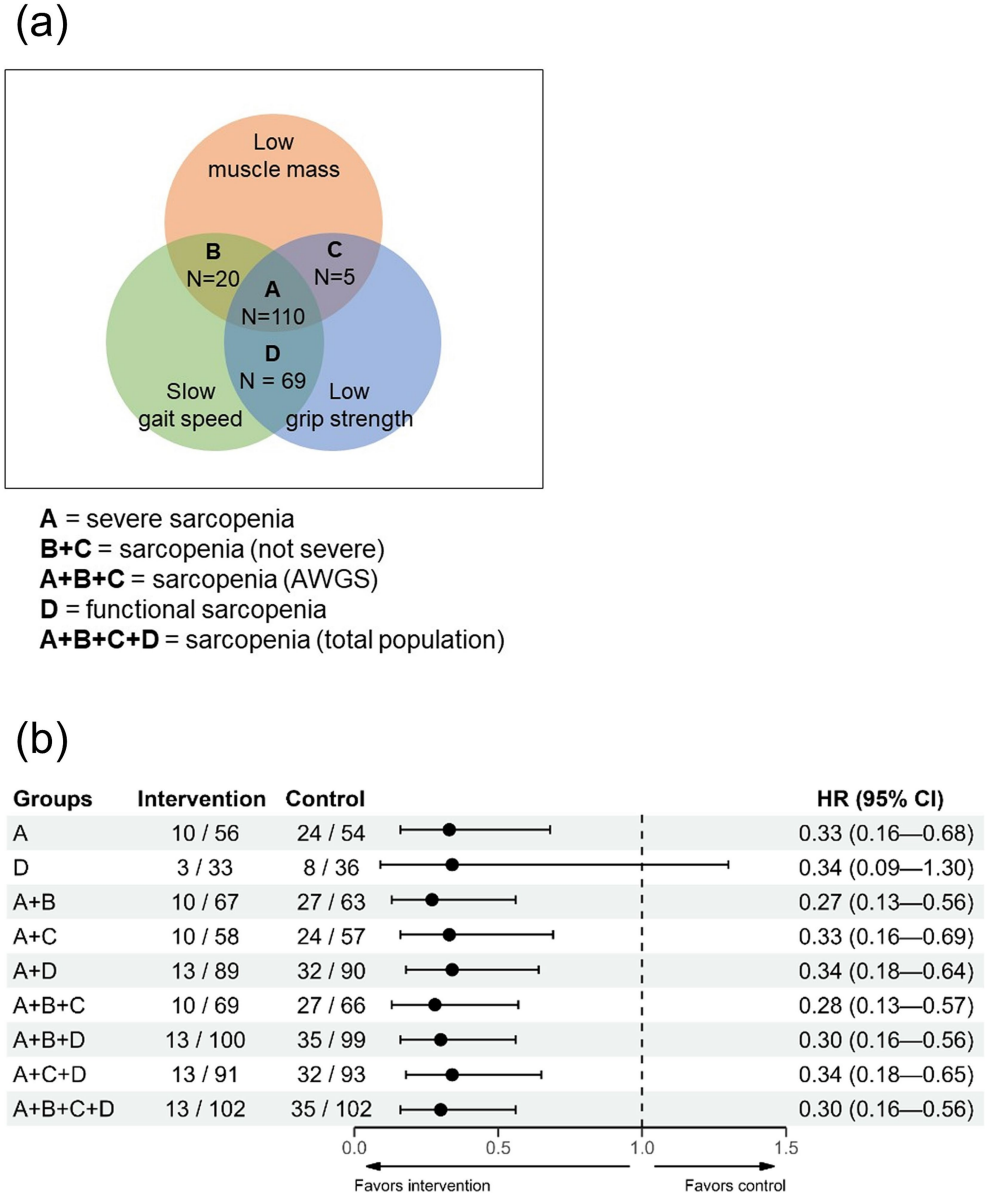
Impact of multicomponent intervention on institutionalization-free survival based on different sarcopenia component combinations (Analysis of Group B or C alone could not be performed due to insufficient data.). **(a)** Venn diagram illustrating study populations and Groups A, B, C, and D. **(b)** Sensitivity analysis examining the effect of multicomponent intervention on institutionalization-free survival across various combinations of Groups A, B, C, and D. AWGS, Asian Working Group for Sarcopenia; CI, confidence interval; SPPB, short physical performance battery.

In addition, we categorized participants into four groups (A, B, C, and D) based on muscle mass, gait speed, and grip strength (Figure 3a). The distribution of participants in each group was as follows: 110 in Group A, 20 in Group B, 5 in Group C, and 69 in Group D. Institutionalization-free survival across all combinations of these four groups was then compared. Notably, the results remained consistent regardless of the combinations, with hazard ratios ranging from 0.27 to 0.34 (Figure 3b).

## 4 Discussion

We found that a 24-week multicomponent intervention, including group exercise, nutritional supplementation, depression management, medication review, and home hazard reduction, was associated with a lower risk of institutionalization and mortality in socioeconomically vulnerable community-living older adults with sarcopenia. Furthermore, the SPPB score and gait speed improved after the intervention and persisted for up to 30 months. The frailty index showed improvement immediately after the intervention but gradually diminished over time. Remarkably, the positive association with institutionalization-free survival persisted irrespective of the combinations of different sarcopenia components. These results suggest that a multicomponent geriatric intervention, as a strategy for addressing frailty, may be effective in managing sarcopenia, regardless of how its components are combined.

The initial definition of sarcopenia focused on the loss of muscle mass associated with aging (38). However, it is now recognized as a systemic and complex condition lacking a single or clear pathophysiology, and no single intervention can completely restore its conditions (2, 39). Furthermore, the consequences of sarcopenia correspond with those of geriatric syndromes and frailty, such as disability, poor quality of life, and increased mortality (16). Therefore, there is a growing perspective that sarcopenia should be considered a geriatric syndrome or physical frailty (13, 15, 16, 26, 40, 41). Recommendations from the World Health Organization (WHO) Guidelines on Integrated Care for Older People (ICOPE), which not only emphasize exercise or nutrition but also polypharmacy, home hazard reduction, or pain management to improve mobility, align with this perspective. Guidelines on sarcopenia from KWGS and Australia and New Zealand highlight the assessment of various components, including falls, cognition, social support, or pain, among others (7, 26). Current approaches extend beyond focusing solely on mobility or muscle, addressing other systemic conditions and patient-centered unmet needs. Our study supports this viewpoint, reinforcing the conceptual alignment between sarcopenia and physical frailty.

Regarding functional sarcopenia, defined as low grip strength and low physical performance with preserved muscle mass according to the KWGS guidelines, our results are noteworthy (26). Functional sarcopenia was previously associated with greater frailty and comparable prognosis compared with sarcopenia (not severe) (13). We demonstrated that a multicomponent intervention is associated with improved outcomes in functional sarcopenia and sarcopenia (AWGS), defined according to the most popular guidelines in the Asian population (Figure 1b) (4). These results suggest that functional sarcopenia should be incorporated into the spectrum of sarcopenia, even with preserved muscle mass. Additional reasons supporting the inclusion of functional sarcopenia into sarcopenia are detailed in the discussion section of our previous study (13).

Our results reinforce the concept that sarcopenia and physical frailty have large similarities and suggest managing sarcopenia in terms of physical frailty, emphasizing a patient-centered and comprehensive approach. First, our results suggest that sarcopenia may benefit from the same intervention strategy with frailty. Second, as mentioned in the previous paragraph, by integrating functional sarcopenia into the sarcopenia spectrum, the operational definition of sarcopenia and physical frailty becomes very similar. Third, characteristics of a continuous concept for sarcopenia, rather than a binary, are suggested by a previous study (42) and our results as described in Figure 3. Fourth, both concepts share similar risk factors and consequences (15, 16). Furthermore, at least in clinical settings, distinguishing between these two concepts and their causal relationship may be impractical and of little importance (15, 16). Further detailed discussions on this topic have been published by various authors (15, 16).

To our knowledge, this study is the first to suggest multicomponent intervention, encompassing various geriatric interventions, such as nutrition, exercise, and deprescribing, can be effective in patients with sarcopenia. Previous studies have shown exercise and nutritional support can be effective in sarcopenia, and well described in a review article (43). A notable example is the result of The Sarcopenia and Physical fRailty IN older people: multi-componenT Treatment strategies (SPRINTT) project, which showed a multicomponent intervention including physical activity and nutritional counseling was associated with a reduction of mobility disability in a multicenter randomized controlled trial with older adults with SPPB score of 3 to 9 points and low appendicular lean mass (44). In addition to nutrition and physical activity, factors such as depression, polypharmacy, and falls have been shown to be associated with sarcopenia (16, 45). However, the effects of psychotherapy or antidepressants, deprescribing, or home hazard reduction have not been well validated. One example is a study indicating that deprescribing was associated with functional recovery and home discharge among older adults with sarcopenia after a stroke (46, 47). We showed that encompassing those approaches with nutrition and exercise was associated with improved outcomes in patients with sarcopenia.

As a post-hoc analysis, this study has inherent limitations, including an increased risk of type I error due to multiple testing, as well as the potential for selection bias and residual confounding (48). While post-hoc subgroup analyses should be interpreted with caution, they can still yield valuable insights—particularly when supported by biological plausibility and a clear clinical rationale, as in our study involving a long-term, comprehensive intervention (49). To address these concerns, we conducted sensitivity analyses, which demonstrated consistent trends across different sarcopenia phenotypes, reinforcing the robustness of our findings. Although exploratory in nature, our results offer meaningful preliminary evidence that may inform the design of future prospective trials targeting this high-risk population.

This study has strength in its long-term follow-up with various geriatric outcomes, and the participants demonstrated higher adherence to the intervention, ranging from 83.7 to 91.3% (25). In addition to the above mentioned limitations of post-hoc analysis, several other limitations should be noted. Firstly, this study was a secondary analysis of a non-randomized trial, and the results should be interpreted with caution. It was conducted in rural areas with limited resources and infrastructure to conduct a randomized controlled trial, a known challenge in community-based interventions for older populations (50). While we attempted to minimize bias using propensity score matching, we acknowledge that these results do not ensure the methodological rigor of a randomized controlled trial. Secondly, generalization is limited since our results were derived from socioeconomically vulnerable older adults in rural areas in Korea. In previous work, we compared the ASPRA cohort with the nationally representative cohort to support external validity (27). Thirdly, since all participants in the intervention group received every aspect of the treatment according to each indication, isolating each component’s impact is impossible. Consequently, we cannot pinpoint which specific element or duration of the program was most effective. Additionally, the multicomponent intervention’s observed benefits could be attributed solely to the nutrition and exercise components, which are already established as effective treatments for sarcopenia (43, 44). Therefore, we believe a randomized controlled trial with a diverse population and various components, intensities, and durations of multicomponent intervention is warranted to validate the effect of such interventions on patients with sarcopenia and implement them in guidelines and public health policy. Finally, approximately 25% of SPPB scores were imputed due to a protocol change early in the study period. While the possibility of bias remains, we used a validated multiple imputation approach incorporating baseline gait speed and available SPPB data from other time points, based on observed longitudinal trends in SPPB (25).

In conclusion, our results demonstrated that a 24-week multicomponent intervention program was associated with a lower incidence of institutionalization and mortality, as well as sustained improvement of physical performance in socioeconomically vulnerable older adults with sarcopenia. Furthermore, this association with institutionalization and mortality rates remained robust across diverse groups, defined by different combinations of sarcopenia components. These results support the perspective of managing sarcopenia as a state of physical frailty, emphasizing a comprehensive geriatric approach as a potential solution for patients with sarcopenia.

## Data Availability

The datasets presented in this article are not readily available because the raw data supporting the conclusions of this article will be made available by the authors upon reasonable request, subject to review. Requests to access the datasets should be directed to onezero2@gmail.com.
