# Supplementary material for: Effect of a multicomponent intervention on institutionalization-free survival in older adults with sarcopenia: a post-hoc analysis
Source: Front Med (Lausanne). 2025 Sep 3;12:1573384. doi: 10.3389/fmed.2025.1573384 (PMC12441017; doi:10.3389/fmed.2025.1573384)
Supplement: Supplementary file 1 [file Table_1.DOCX]

**Effect of multicomponent intervention in community-dwelling older adults with sarcopenia: A post-hoc analysis**

**Supplementary Data**

**Table of Contents**

- **eFigure 1.**  Study Design Flowchart
- **eTable 1.**  List of components included in the 47-item Frailty Index

**eFigure 1.** Study Design Flowchart


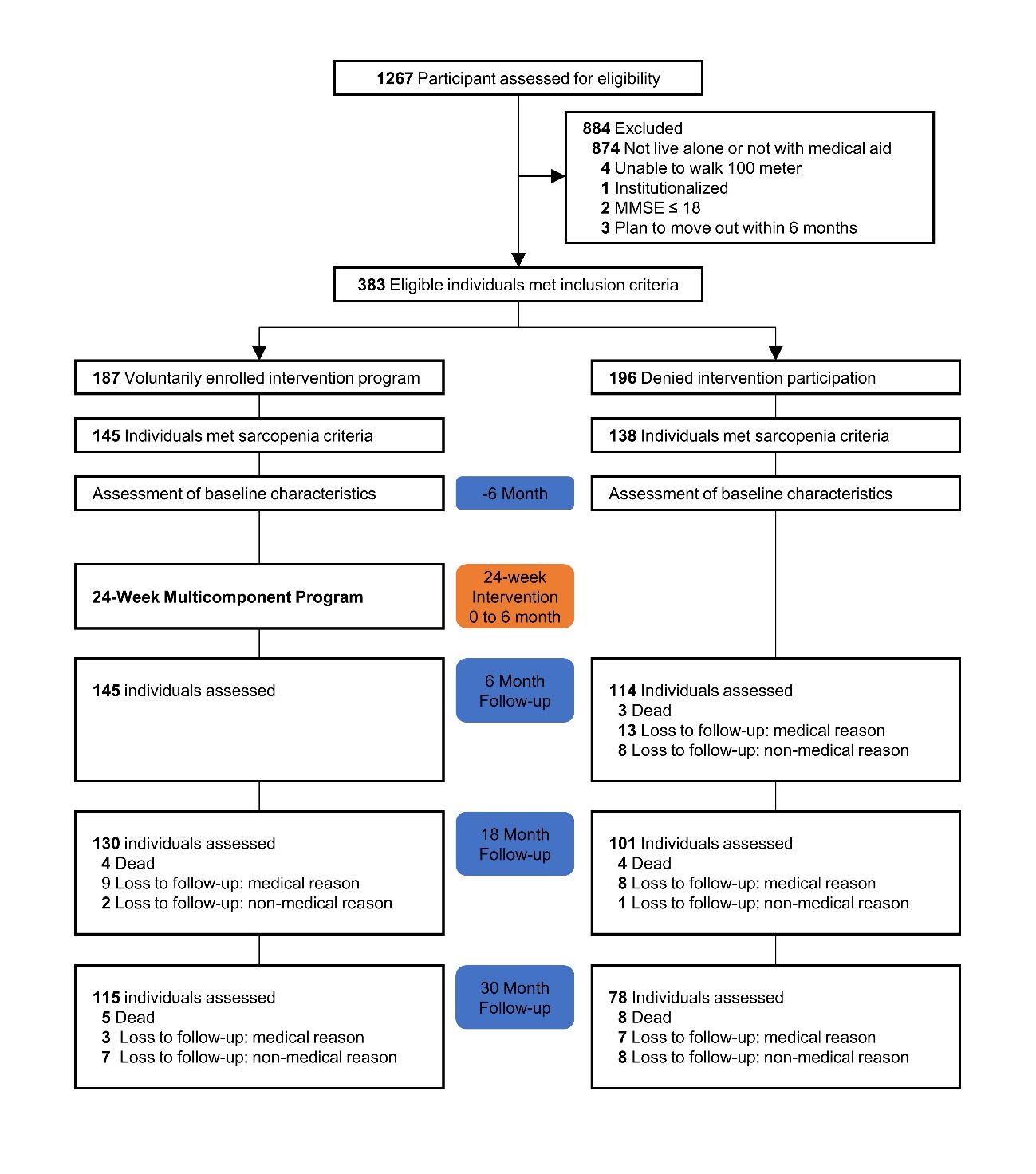


**eTable 1.** List of components included in the 47-item Frailty Index

| **Category** | **Deficit Items** |
| --- | --- |
| Medical comorbidities  (14 items) | - Hypertension - Diabetes - Cancer (other than a minor skin cancer) - Chronic lung disease - Heart attack - Congestive heart failure - Angina - Asthma - Arthritis - Stroke - Kidney disease - Dementia - Falls   Constipation/IBS |
| Self-reported functional status and disability  (21 items) | - Dressing - Washing face, hair, or toothbrushing - Bathing or shower - Feeding - Getting in and out of bed - Toileting - Fecal and urinary continence - Fatigue - Resistance - Ambulation - Pain: Question from EQ-5D - Using telephone - Using transportation - Shopping - Preparing own meals - Housework - Taking own medications - Managing money - Grooming - Doing laundry - Going out short distances without using transportation |
| Physical performance  (5 items) | - Low physical activity: International Physical Activity Questionnaire (IPAQ) – Short Form (below 20 percentile in KNHANES) - Dominant handgrip strength: < 26 kg for men and < 18 kg for women - SPPB score: balance < 3 (1point)   repeated chair stand < 3 (1point)  gait speed <3 (1point) |
| Mood  (3 items) | - Center for Epidemiologic Studies Depression Scale >20 - Exhaustion: Positive answer to either of the following statements: “I felt that everything I did was an effort” or “I could not get going.” Participants who answered “ a moderate amount of the time (3–4 days)" or “ most of the time” to either of these questions were considered as exhaustion. - Anxiety: Question from European Quality of Life-5 Dimensions (EQ-5D) |
| Cognition (1 item) | - Mini-Mental State Examination Dementia Screening <24 |
| Nutritional status (1 item) | - Malnutrition: Mini Nutritional Assessment-Short Form score ≤11 |
| Polypharmacy (1 item) | - Number of medications ≥5 |
| Social interaction (1 item) | - Social Frailty score ≥2 |

Source: Oh G, Lee H, Park CM, Jung H-W, Lee E, Jang I-Y, et al. Long-term effect of a 24-week multicomponent intervention on physical performance and frailty in community-dwelling older adults. Age and Ageing. 2021;50:2157-66.
